# Physical Activity and Accomplishment of Recommendations in University Students with Disabilities: A Longitudinal Study

**DOI:** 10.3390/ijerph18115540

**Published:** 2021-05-22

**Authors:** Miquel Pans, Joan Úbeda-Colomer, Javier Monforte, José Devís-Devís

**Affiliations:** 1 AFES Research Group, Departament d’Educació Física i Esportiva, FCAFE, Universitat de València, 46010 València, Spain; miquel.pans@uv.es (M.P.); joan.ubeda-colomer@uv.es (J.Ú.-C.); jose.devis@uv.es (J.D.-D.); 2 Department of Sport and Exercise Sciences, Durham University, 42 Old Elvet, Durham DH1 3HN, UK

**Keywords:** physical activity, disability, students, university, cohort

## Abstract

University settings are socio-environmental contexts that can reduce health disparities in students with disabilities. Therefore, the aim of this study was twofold: (a) to examine the longitudinal physical activity (PA) changes of Spanish university students with disabilities during a three-year period; and (b) to identify the accomplishment of the World Health Organization’s PA recommendations in this period. A three-year follow-up cohort study was conducted on 355 university students with disabilities (172 men, 183 women). The participants completed an electronic survey on PA after which a descriptive analysis, longitudinal (Wilcoxon tests) and cross-sectional pairwise comparisons (Mann–Whitney U and Kruskal–Wallis tests) were performed on non-normal data. The results show no significant PA changes during the three-year period. The cross-sectional comparisons between the waves presented a reduction in vigorous PA according to sex and similar values by age, origin of disability, and socioeconomic status. A global reduction of 0.6% was found in achieving the recommendations between the waves. We also found an increase of 5.3% in the participants classified as overweight–obese during this period. The findings offered in this study have important implications for university disability care services and sports services. University policies should focus on rethinking PA and sports programs for students with disabilities.

## 1. Introduction

Physical activity (PA) offers multiple health benefits for people with disabilities. For example, it improves bone density and muscle mass, reduces pain and the risk of chronic disease, and contributes to maintaining body weight. It also helps to reduce depression, positively affects mood, and has the potential to improve physical function, wellbeing, and community inclusion [1,2,3,4]. Despite these benefits, the epidemiological data show that adults with disabilities are less active and present higher rates of chronic disease than the general population [5,6,7]. In fact, compared with their non-disabled counterparts, research shows lower PA prevalence in different disability groups, including people with intellectual and mobility disabilities and chronic illness [2,8,9,10]. Adults with disabilities are less likely to meet the World Health Organization’s (WHO) PA recommendations (75 min/week of vigorous or 150 min/week of moderate aerobic PA or an equivalent combination of 600 Metabolic Equivalent of Task (MET) min/week) than adults without disabilities [6]. The studies carried out on particular disability groups indicated that the different prevalence in meeting the recommendations ranged from 42% in older adults with diabetes mellitus [10] to 0% in individuals with intellectual disabilities [9]. Furthermore, when two or more disabilities are experienced, the level of PA and likelihood of meeting recommendations is alarmingly reduced [3,11].

Apart from the particular features of each disability, many different personal, socioeconomic, and environmental factors can affect the PA engagement of people with disabilities, thus exacerbating health inequalities [12]. In this regard, people with disabilities are more likely to be obese and to have diabetes, high cholesterol, and hypertension than non-disabled people [13,14]. People with disabilities also show lower educational attainment, lower earnings, a higher rate of unemployment, and are more likely to receive welfare benefits than non-disabled people [15,16]. Faced with this situation, it is crucial that institutions and public policies generate healthy and active environments. University settings are among the contexts that can reduce health disparities in students with disabilities since they provide facilities and material resources as well as staff for promoting healthy lifestyles and social wellbeing on and off campus [17]. The “Healthy Universities” initiative can be helpful in this regard because it runs campaigns and activities for PA promotion [18]. Most Spanish universities have adhered to this initiative, establishing the Spanish Network of Healthy Universities (see https://www.unisaludables.es/es/, accessed on 8 May 2021).

In sharp contrast, different studies suggest that university students with and without disabilities are not achieving the recommendations stated before. For instance, US students’ PA has been found to decrease in the transition from high school to university [19]. Among Spanish university students, 47.7% do not meet the recommended PA levels or its equivalent in energy expenditure (600 MET min/week), with women being below this percentage (41.7%) [20], and 51.39% of university students spending less than 30 min daily on moderate to vigorous physical activity (MVPA) [21]. The percentage of university students classified as overweight–obese was found to increase throughout their university careers in both males and females [22]. Some studies indicate that PA participation among university students with disabilities seems to be poorest. For example, the use of campus PA facilities of US students with disabilities was significantly lower than their non-disabled peers [23]. A recent study conducted in Spanish universities found that 63.1% of these students did not meet any of the WHO’s PA recommendations for achieving health benefits. Particularly, 72.2% did not meet the recommendation of 75 min/week of vigorous PA and 80.3% did not meet the recommendation of 150 min/week of moderate PA [11]. However, no significant differences were found in the PA rates between US university students with and without disabilities, although these rates were significantly higher in males with disability than females [24].

According to the previous review, further research is still necessary to determine how time, personal, and disability factors affect the PA of university students with different disabilities and origins in order to improve this behavior and enhance its multiple benefits. Longitudinal studies are especially important for knowing how healthy initiatives on PA are developed due to the lack of information available on the changes over time within university settings. In this context we therefore carried out the present prospective cohort study with the aim of (a) examining the longitudinal PA changes of Spanish students with disabilities over a three-year period (2016–2019) and (b) identifying their percentage compliance with the WHO’s PA recommendations for this population in this period.

## 2. Materials and Methods

### 2.1. Study Design and Participants

**Figure 1:**
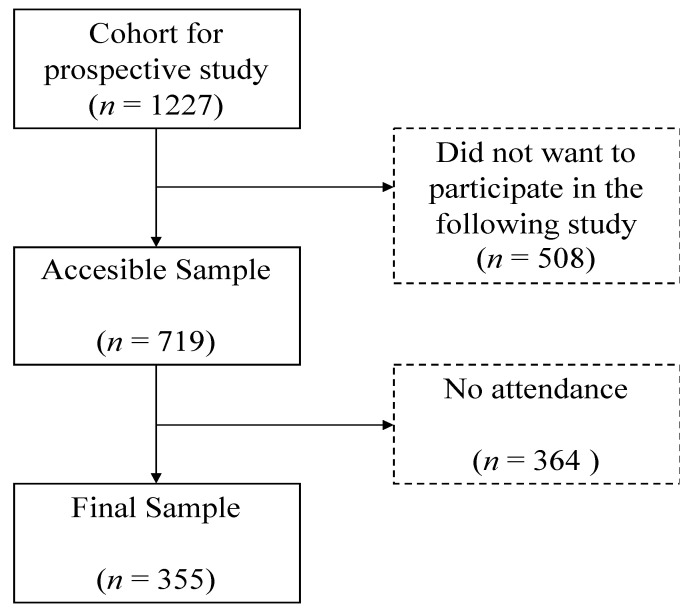
Flow diagram of the process of obtaining the final sample of the study.

University students with disabilities belonging to 55 Spanish Universities, most of them members of the Spanish Network of Healthy Universities, participated in a prospective cohort study. This sample was accessed in Wave I via the universities’ disability care services because they prevented us from directly assessing students due to data protection policies. Later, in Wave II, those who wanted to continue in the study were contacted through their personal university email addresses, which were voluntarily provided. Each student with a disability received a link for an online survey on LimeSurvey (2.05+) free software (LimeSurvey GmbH, Hamburg, Germany). Only two of them manifested problems of accessibility and the survey was completed by phone. The cohort was defined by students who had participated in a previous cross-sectional representative study [11] in 2016 (Wave I) and wanted to continue participating in the study three years later (Wave II). Of the original cohort of 1227 students with disabilities that participated in Wave I, 719 remained as the accessible cohort for Wave II. From the remained sample, 364 did not participate in the data collection for unknown reasons. Eventually, 355 university students with disabilities (172 men and 183 women) participated in both Wave I and Wave II. The final sample was 49.4% of the accessible cohort (see Figure 1).

Table 1 shows the key sample characteristics. Prior to administering the survey, all the procedures and materials were approved by the Ethics Committee of the University of Valencia (Code: H1436947544660). All the participants were emailed a link to an informed consent form that explained the conditions of participation (e.g., confidentiality, anonymity, right to refuse or abandon). To access the full survey, they clicked on a box giving their informed consent to participate.

### 2.2. Measures

#### 2.2.1. Physical Activity

Overall and particular PA domains (i.e., vigorous PA, moderate PA, and walking intensity PA) were measured by the International Physical Activity Questionnaire-short form (IPAQ). The IPAQ was created by Craig et al. [25] and has been used worldwide to collect PA data. This questionnaire was modified to be more inclusive for assessing adapted physical activity, as in Rosenberg et al. [26] (e.g., vigorous activities including wheelchair racing or handbiking, moderate activities and walking activities including wheeling) and was applied recently in Spanish studies [11,27]. In addition, when walking, moderate and vigorous PA exceeding 180 min was re-coded to 180 min.

Participants were classified as “meet recommendations” when they reported at least 150 min of moderate or 75 min of vigorously intense aerobic PA per week, according to WHO [7,28], or an equivalent combination of 600 MET min/week. This equivalent combination is the criterion we used for comparative purposes with WHO’s PA recommendations and the wider literature, as stated by Hallal et al. [29] and used in disability studies [26,30].

#### 2.2.2. Sociodemographic Variables

Sociodemographic data were collected through several questions at the beginning of the survey (sex, age, disability condition, and origin of disability). Sex was classified according to two categories. Age was an open question divided into three categories using percentiles 33 and 66. Disability condition was determined by the participants’ response to questions pertaining to physical disability, mental disorder, sensory disability, and chronic illness, and were classified as participants with a single or multi-disability. The origin of the disability was a binomial question comprising two options: congenital or acquired.

#### 2.2.3. Weight Status

Weight and height were collected in the survey as perceived data for subsequent calculation of body mass index (BMI): weight (kg)/height (cm^2^). The BMI cut-off values were those indicated by the WHO [31]. The participants were then grouped into two weight categories: underweight–normal range and overweight–obese.

##### 2.2.4. Socioeconomic Status

Socioeconomic status (SES) was given through another open question and divided into three categories using percentiles 33 and 66 (i.e., low, middle, high), following the same criteria as in previous studies [32].

### 2.3. Statistical Analysis

Statistical analyses were performed on SPSS Version 26.0 software (SPSS Inc., Chicago, IL, USA) with alpha set at *p* < 0.05. As the Kolmogorov–Smirnov test showed a non-normal distribution, descriptive statistics were expressed as medians and interquartile ranges (IQR). Wilcoxon tests were used for the repeated measures of inferential statistics to analyze longitudinal changes, and Mann–Whitney U and Kruskal–Wallis tests were used for cross-sectional inference in Waves I and II. To detect any bias in the participants’ data, comparisons were made between those who participated in Wave II and those who did not, which confirmed that the data were not biased in any of the variables of interest.

## 3. Results

### 3.1. Longitudinal Changes

The 355 participants who completed the follow-up study presented similar sociodemographic characteristics to those who withdrew. Table 2 shows the descriptive statics of all PA domains in both waves. The values of overall, vigorous, moderate, and walking intensity PA were similar over the three-year period. Wilcoxon tests revealed no statistically longitudinal significant differences in all the PA domains between Waves I and II (*p* > 0.001). Although the vigorous PA domain had the biggest reduction, it was not statistically significant compared with the changes in the other domains.

In order to identify any changes in PA by variables of interest (i.e., sex, age, disability condition, origin of disability, weight status, and SES), cross-sectional comparisons were determined between waves (see Table 3). Kruskal–Wallis and Mann–Whitney U tests revealed a statistically significant difference in PA by age, disability condition, and weight status in Wave I. The youngest group of participants reported higher overall PA values than the middle group (*p* < 0.006), and this group also scored higher than the middle (*p* < 0.002) and the older (*p* < 0.001) groups in vigorous PA. Students with a single disability condition reported higher values in vigorous (*p* < 0.006) and moderate (*p* < 0.007) PA than those with multi-disability conditions. Those students with underweight–normal range status reported significantly higher values in overall PA (*p* < 0.001), vigorous PA (*p* < 0.015), and walking (*p* < 0.011) than those with overweight–obese status.

Similar results were found in Wave II, where Kruskal–Wallis and Mann–Whitney U tests showed statistically significant differences by sex, age, disability condition, and weight status. The male and younger group of students reported higher values in vigorous PA than their female counterparts (*p* < 0.010) and the middle (*p* < 0.015) and older (*p* < 0.017) groups, respectively. Those with a single disability condition reported higher values in moderate PA than those with a multi-disability condition (*p* < 0.032). Students with underweight–normal range status reported higher values in overall (*p* < 0.005), vigorous (*p* < 0.004), and moderate PA (*p* < 0.021) than those with overweight–obese status.

### 3.2. Accomplishment of PA Recommendations

Table 4 presents the sociodemographic characteristics of the participants according to compliance with the WHO’s PA recommendations and their weight status in both waves. An overall reduction of 0.6% in compliance with the recommendations was found between waves in the three-year period (41.4% in Wave I and 40.8% in Wave II). A similar reduction was detected by sex (0.6% in men and 0.5% in women). A moderate decrease of 0.8% and 1.7% was also noted among the participants in the 18–35 and 36–45 age ranges, while an increase of 0.8% was seen in the older range. There was a decrease of 4% in the participants’ accomplishment with single disability condition and an increase of 10.7% among those with multi-disabilities. According to the origin of the disability, an increase of 0.8% was noticed among participants with congenital disabilities and a decrease of 1.3% in accomplishment in people with acquired disabilities. Participants with low SES also increased their compliance by 3%, while a decrease appeared in university students with middle (1.5%) and high (1.7%) SES.

As can be seen in Table 4, the percentage of people classified as overweight–obese increased in the whole sample from Wave I to Wave II (5.3%) and also according to the variables of interest regardless of whether they increased or reduced their compliance with the WHO’s PA recommendations.

## 4. Discussion

This is the first longitudinal study to examine PA participation and compliance with the WHO’s PA recommendations in a sample of Spanish university students with disabilities over a three-year period. The study’s main finding was that there were no significant changes in any of the domains of the participants’ PA levels. This may suggest that PA promotion policies, such as the “Healthy Universities” initiative [18], need to be reviewed and that more efforts are required from academic institutions to substantially improve these issues. Otherwise, Spanish universities are under the risk of becoming non-proactive entities for improving their students with disabilities PA and health as required by the “Healthy Universities” initiative. The socio-environmental factors both within and outside the university should therefore be considered as a way to strengthen promotion strategies for improving healthy lifestyles among university students with disabilities by increasing their PA participation.

The comparison between waves shows that vigorous PA values are lower in Wave II than Wave I with respect to sex, with significant differences between men and women in Wave II that were not found in Wave I. There was a considerable reduction in women’s vigorous PA in the three-year period compared with a slight decrease in men. This is consistent with previous studies in which men reported higher values of PA than women among university students with disabilities [11,24] and more generally among people with disabilities [33,34]. This is probably due to the relevance of the barriers to women’s engagement in PA. According to Úbeda-Colomer et al. [35], women students with disabilities experience more intrapersonal barriers (e.g., motivation, fatigue, pain) than their male peers. Moreover, women with spinal cord injuries show less confidence in overcoming PA barriers and less control in these practices than their male peers [36].

The results of the present study show similar values in the different PA domains between waves by age, origin of disability, and SES, with significant differences in vigorous PA according to age and no differences by origin and SES in either wave. These results allow us to suggest that, generally, there are no changes in PA by these variables over a three-year period. Even so, the youngest group of students is the most active group in both waves, compared with middle and older groups, as has also been observed in previous cross-sectional studies with university students with disabilities and people with spinal cord injuries [11,37]. This is probably due to family unavailability for supporting them and to perceived risks of injuries or falls during adult and older life, as indicated elsewhere [2,38,39]. No differences by origin of disability and SES in both waves indicate that PA is not affected by these variables in this population, although some studies found that those with high incomes have more access to PA participation [40].

Nevertheless, significant differences are observed in moderate PA in both waves by the number of disability conditions; those with multi-disability conditions report lower values in this PA domain. Of additional interest, comparing the descriptive statistics between waves shows that moderate PA decreases among participants with a single disability while it increases among those with multi-disability between Wave I and Wave II. Significant differences in vigorous PA are also shown in Wave I, in which those with multi-disability conditions report lower values, even though these differences do not appear in Wave II. Taken together, all these observations suggest that moderate PA may be easier to access for people with disabilities than vigorous PA, especially among those with multi-disabilities.

Regarding cross-sectional comparisons of PA by weight status, it can be seen that overall, vigorous, and walking PA present significant differences between underweight–normal and overweight–obese persons in Wave I and overall vigorous and moderate PA in Wave II. The underweight–normal participants reduce their PA values in all domains, except for a small increase in the median of moderate PA over the three-year period. On the other hand, the overweight–obese participants increase in overall, moderate, and walking PA domains during the same period. This seems to be a counterintuitive result because the overweight–obese are those who clearly increase overall PA. This result has been found previously in other Spanish populations such as adolescents [41], probably because these persons most in need of PA are more conscious of its health benefits and increase their PA engagement.

In both waves, the percentages of compliance with the WHO’s PA recommendations for the whole sample are lower than the 52.3% found in the general population of Spanish university students [20]: 10.9% less in Wave I and 11.5% less in Wave II. This result is consistent with previous studies that found that adults with disabilities are less active and less likely to meet WHO’s recommendations than their counterparts with no disabilities [5,11,42,43]. The percentages of accomplishment in both waves of the present study are also lower than the 42% found among older adults with diabetes mellitus [10], although they are higher than individuals with intellectual disabilities, who showed no compliance at all [9].

A decrease in the accomplishment of the WHO’s PA recommendations is observed according to sex, the 18–35 and 36–45 age ranges, middle and high SES, and among people with single disability and acquired disability. Conversely, the older group of university students with disabilities and those with multi-disabilities and low SES increase the percentage of accomplishment. It seems that the older participants, for whom PA is crucial [41], are more conscious of its health benefits and increase their PA engagement. However, the rise in the percentages of overweight–obese participants that did not meet the recommendations in the three-year period appears to contradict the PA increase shown by this group. That is, overweight–obese participants increased their PA over the three-year period, mostly due to an increase in moderate and walking intensity PA, but the percentage of overweight–obese people that did not meet the recommendations also increased. All the same, the rise in the percentages of overweight–obese participants in the three-year period (from 41.3% to 51.6%) is in line with the contributions that found an increase in weight among the general population of university students [22]. In general, these findings suggest that an increase based on moderate PA and especially in walking wheelchair rolling intensity, the most common activity among adults with disability [44], might not be enough to control or reduce weight status and obtain substantial health benefits in persons with disabilities, as previously indicated [3]. More allies are required to affect their overweight–obesity status [45]. Other personal and socio-environmental factors in the lives of the participants of this study [46] would help us understand the increase of overweight–obese percentages. For instance, a previous study mentions organizational barriers (e.g., lack of adapted programs, economic cost) at the university as key issues in this regard [33]. However, more efforts in developing PA promotion strategies are still required to achieve higher percentages of accomplishment with the WHO’s PA recommendations.

Finally, the current study has some potential limitations that warrant consideration. First, the non-normal distribution of the data required the use of non-parametric tests and prevented the implementation of more sophisticated analyses [47]. Non-parametric methods may have less statistical power than parametric ones. In addition, the analyses performed cannot explore possible interactions between the variables (e.g., if relevant sociodemographic variables could be exerting a moderating effect on potential changes in PA over time). However, since this is the first longitudinal study to examine PA changes in university students with disabilities, the results that we offer represent a relevant contribution and some keys for the promotion of PA in this group are provided. Secondly, due to strict data protection policies from the universities it was not possible to administer face-to-face surveys. Data were collected online through the Spanish university disability services, and this reduced the researcher’s control of the process and made it difficult to address the participants’ doubts. However, we provided clear instructions and the participants were encouraged to read all the questions carefully. Finally, although the use of self-reported measures is not exempt from bias, the IPAQ has been used worldwide and allows national and international comparisons [25,34].

## 5. Conclusions

This paper shows that PA participation and the compliance with the WHO’s recommendations (75 min/week of vigorous or 150 min/week of moderate aerobic PA or an equivalent combination of 600 MET min/week) is still very low among university students with disabilities. These results can raise awareness of physical inactivity and inform future PA and sport programs for university students with disabilities. Therefore, it is necessary to put further effort into facilitating the accomplishment of these recommendations at universities, as well as to consider personal and socioenvironmental factors in order to make universities truly healthy environments.

## Figures and Tables

**Table 1:** Sociodemographic characteristics of the sample (*n* = 355).

Variable	*n*	% Total
Sex		
Male	172	48.5
Female	183	51.5
Missing	0	0
Age		
18–35	117	33
36–45	119	33.5
>45	119	33.5
Missing	0	0
Disability condition		
Single disability	271	76.3
Multi-disablility	84	23.7
Missing	0	0
Origin of disability		
Congenital	126	35.5
Acquired	229	64.5
Missing	0	0
Weight status		
Underweight–Normal range	171	48.2
Overweight–Obese	182	51.3
Missing	2	0.6
Socieoconomic Status		
Low	122	34.4
Middle	108	30.4
High	119	33.5
Missing	6	1.7

**Table 2:** Physical activity values (MET minutes/week) (*n* = 355) in Wave I and II and Wilcoxon test for significant changes.

	Wave I				Wave II				Wilcoxon Test
Physical Activity Domains	M	SD	Med	IQR	M	SD	Med	IQR	*p*-Value
Overall	1838.25	2203.70	1215	2118	1824.58	2152.06	1200	1950	0.909
Vigorous	701.97	1384.72	0	960	632.22	1320.13	0	720	0.097
Moderate	370.30	740.19	0	480	357.55	713.27	0	480	0.922
Walking	765.97	1009.63	462	1188	834.80	1091.33	462	1386	0.371

M = mean; Med = median; IQR = interquartile range; SD = standard deviation.

**Table 3:** Comparison of physical activity (MET minutes/week) by variables of interest in Wave I and Wave II.

Physical Activity			Wave I				Wave II		
		Overall	Vigorous	Moderate	Walking	Overall	Vigorous	Moderate	Walking
	Sex								
M (SD)	Men	1890 (2086.57)	836.27 (1504.86)	363.13 (745.7)	691.18 (897.46)	1945 (2160.9)	809.3 (1525.56) *	355.12 (664.9)	780.58 (1013.55)
	Women	1789.05 (2313.02)	575.74 (1252.5)	377 (736.97)	836.27 (1102.58)	1711.41 (2143.46)	465.79 (1073) *	359.85 (757.76)	885.77 (1160.18)
Med (IQR)	Men	1386 (2259)	0 (1110)	0 (480)	396 (1027.13)	1331.75 (2225.63)	0 (960) *	0 (480)	433.95 (1336.5)
	Women	1127.5 (2188.5)	0 (560)	0 (480)	462 (1188)	1053 (1923)	0 (480) *	0 (480)	462 (1386)
	Age								
M (SD)	18–35	2393.07 (2743.27) *	1149.05 (1882.4) *	446.66 (797.75)	787.34 (1023.93)	2171.51 (2433.97)	931.28 (1673.65) *	414.52 (721.18)	825.70 (1149.59)
	36–45	1507.38 (1959.18) *	443.36 (927.91) *	320 (729.37)	744.02 (1018.65)	1510.82 (1917.55)	460.16 (1113) *	284.63 (680.11)	766 (1050.06)
	>45	1633.45 (1698.66)	521 (1053) *	345.54 (690.70)	766.90 (994.38)	1797.25 (2043.02)	510.25 (1049.90) *	374.45 (737.27)	912.54 (1386)
Med (IQR)	18–35	1440 (2367) *	240 (1740) *	0 (720)	462 (1386)	1386 (2750.25)	0 (1380) *	8 (600)	346.50 (1353)
	36–45	924 (1878) *	0 (720) *	0 (360)	462 (924)	990 (1712)	0 (480) *	0 (360)	422.40 (1188)
	>45	1155 (2454)	0 (480) *	0 (480)	396 (1386)	1275 (1900.50)	0 (720) *	0 (500)	594 (1386)
	Disability condition								
M (SD)	Single	1975 (2369.23)	799.11 (1496) *	413 (781.69) *	763.07 (984.92)	1796.83 (2151.15)	640.73 (1313.81)	375.89 (698.95) *	780.20 (1064.15)
	Multi	1394.83 (1480.44)	388.57 (1496) *	230 (568.57) *	775.30 (1091.70)	1914.12 (2165.50)	604.76 (1347.93)	298.38 (758.99) *	1010.97 (1163.95)
Med (IQR)	Single	1314 (2118)	0 (1080) *	0 (560) *	462 (1155)	1188 (1846)	0 (720)	0 (480) *	422.40 (1188)
	Multi	723.75 (1881)	0 (360) *	0 (240) *	239.25 (1386)	1386 (2454)	0 (720)	0 (175) *	742.50 (1386)
	Origin of disability								
M (SD)	Congenital	1993.36 (2309.77)	809.52 (1591.64)	369.84 (720.75)	814 (989.96)	1904.28 (1276.25)	844.44 (1691.02)	316.47 (516.08)	743.36 (962.39)
	Acquired	1752.90 (2143.48)	642.79 (1256.31)	370 (752.23)	739.54 (1021.47)	1780.74 (2149.67)	515.46 (1048.58)	380.15 (801.53)	885.12 (1155.10)
Med (IQR)	Congenital	1261.50 (2081.25)	0 (960)	0 (480)	495 (1386)	1276.25 (2162)	0 (960)	0 (480)	409.20 (1386)
	Acquired	1215 (2198.25)	0 (960)	0 (480)	396 (1039.50)	1188 (1884.75)	0 (640)	0 (480)	462 (1386)
	Weight Status								
M (SD)	Underweight–Normal	2288.51 (2625.65) *	907.93 (1668.37) *	457.88 (892.79)	922.69 (1142.92) *	2169.65 (2466.62) *	848.42 (1616.09) *	401.70 (781.11) *	919.52 (1151.59)
	Overweight–Obese	1336.05 (1446.73) *	470 (921.83) *	271.16 (502.35)	594.70 (803.71) *	1512.81 (1764.63) *	436.04 (931.74) *	320 *	756.76 (1032.16)
Med (IQR)	Underweight–Normal	1426 (2360.50) *	0 (1440) *	0 (480)	495 (1386) *	1386 (2304) *	0 (960) *	8 (480) *	660 (1386)
	Overweight–Obese	840 (2016) *	0 (480) *	0 (480)	231 (792) *	960 (1890) *	0 (480) *	0 (390) *	396 (1336.5)
	Socioeconomic Estatus								
M (SD)	High	1784.64 (2207.10)	666.66 (1322.74)	335.95 (599.15)	782 (1081.75)	1941.84 (2484.67)	610 (1214)	448.64 (830.86)	883.10 (1203.53)
	Middle	1657.91 (1894.74)	638.44 (1200.77)	291.16 (576.31)	728.30 (991.15)	1633.72 (1918.95)	587.90 (1223.73)	301.70 (677.94)	744.16 (1021.35)
	Low	2106.81 (2521.55)	790.33 (1614.53)	495.33 (971.02)	821.15 (989.67)	1968.10 (2121.31)	707.66 (1514.53)	350.13 (658.29)	910.30 (1078.91)
Med (IQR)	High	1039 (2502)	0 (720)	0 (480)	247.50 (1386)	1200 (2530.40)	0 (960)	100 (560)	396 (1386)
	Middle	1182 (2369)	0 (960)	0 (480)	396 (1014.75)	1140 (1685)	0 (720)	0 (300)	462 (1113.75)
	Low	1386 (2039.25)	0 (960)	0 (480)	495 (1336.50)	1386 (1963.13)	0 (780)	0 (530)	693 (1386)

* significant at *p* < 0.05. IQR = interquartile range; SD = standard deviation.

**Table 4:** Percentages of participants’ accomplishment of WHO’s PA recommendations and their weight status by wave and individual characteristics (*n* = 355).

	Wave I				Wave II			
	Meet PA	Not Meet PA	Under/ Normal Weight	Overweight/ Obese	Meet PA	Not Meet PA	Under/Normal Weight	Overweight/ Obese
Whole sample	41.4	58.6	53.7	46.3	40.8	59.2	48.4	51.6
Sex								
Men	46.5	53.5	46.5	53.5	45.9	54.1	40.4	59.6
Women	36.6	63.4	60.4	39.6	36.1	63.9	56	44
Age								
18–35	50.4	49.6	69	31	49.6	50.4	65.8	34.2
36–45	35.3	64.7	53.8	46.2	33.6	66.4	47.9	52.1
>45	38.7	61.3	38.7	61.3	39.5	60.5	31.6	68.4
Disability condition								
Single	44.6	55.4	54.8	45.2	40.6	59.4	51.5	48.5
Multi	31	69	50	50	41.7	58.3	38.6	61.4
Congenital/acquired								
Congenital	44.4	55.6	62.4	37.6	45.2	54.8	56.8	43.2
Acquired	39.7	60.3	48.9	51.1	38.4	61.6	43.9	56.1
Socioeconomic Estatus								
Low	41.6	58.4	69	31	44.6	55.4	54	46
Middle	39.5	60.5	53.8	38.7	38	62	41.4	58.6
High	42.5	57.5	58	42	40.8	59.2	52.5	47.5
Missing								

Meet PA: meet the WHO’s PA recommendations.

## Data Availability

The data presented in this study are available on request from the corresponding author. The data are not publicly available due to ethical reasons.
